# Region-specific role of growth differentiation factor-5 in the establishment of sympathetic innervation

**DOI:** 10.1186/s13064-016-0060-3

**Published:** 2016-02-15

**Authors:** Gerard W. O’Keeffe, Humberto Gutierrez, Laura Howard, Christopher W. Laurie, Catarina Osorio, Núria Gavaldà, Sean L. Wyatt, Alun M. Davies

**Affiliations:** School of Biosciences, Cardiff University, Museum Avenue, Cardiff, CF10 3AT UK; Dept. Anatomy/Neuroscience and Biosciences Institute, UCC, Cork, Ireland; Current address, School of Life Sciences, University of Lincoln, Brayford Pool, Lincoln, LN6 7TS UK; Current address, MRC Centre for Developmental Neurobiology, King’s College London, New Hunt’s House, 4th Floor, Guy’s Hospital Campus, London, SE1 1UL UK; Current address, SOM Innovation Biotech SL, c/Baldiri Reixac 4, 08028 Barcelona, Spain

**Keywords:** Sympathetic neuron, Axon growth, Innervation, GDF5

## Abstract

**Background:**

Nerve growth factor (NGF) is the prototypical target-derived neurotrophic factor required for sympathetic neuron survival and for the growth and ramification of sympathetic axons within most but not all sympathetic targets. This implies the operation of additional target-derived factors for regulating terminal sympathetic axon growth and branching.

**Results:**

Here report that growth differentiation factor 5 (GDF5), a widely expressed member of the transforming growth factor beta (TGFβ) superfamily required for limb development, promoted axon growth from mouse superior cervical ganglion (SCG) neurons independently of NGF and enhanced axon growth in combination with NGF. GDF5 had no effect on neuronal survival and influenced axon growth during a narrow window of postnatal development when sympathetic axons are ramifying extensively in their targets in vivo. SCG neurons expressed all receptors capable of participating in GDF5 signaling at this stage of development. Using compartment cultures, we demonstrated that GDF5 exerted its growth promoting effect by acting directly on axons and by initiating retrograde canonical Smad signalling to the nucleus. GDF5 is synthesized in sympathetic targets, and examination of several anatomically circumscribed tissues in *Gdf5* null mice revealed regional deficits in sympathetic innervation. There was a marked, highly significant reduction in the sympathetic innervation density of the iris, a smaller though significant reduction in the trachea, but no reduction in the submandibular salivary gland. There was no reduction in the number of neurons in the SCG.

**Conclusions:**

These findings show that GDF5 is a novel target-derived factor that promotes sympathetic axon growth and branching and makes a distinctive regional contribution to the establishment of sympathetic innervation, but unlike NGF, plays no role in regulating sympathetic neuron survival.

## Background

Nerve growth factor (NGF) is the prototypical target-derived neurotrophic factor on which the foundations of neurotrophic theory are based [1]. In the developing peripheral nervous system, postganglionic sympathetic neurons and the majority of sensory neurons depend for their survival on a supply of NGF synthesized in their targets [2, 3]. While target-derived NGF plays no role in guiding axons to their targets during development [4], it acts locally on axon terminals within the target field to promote growth and branching within most but not all targets of NGF dependent neurons [5, 6]. This implies the operation of additional target-derived factors that regulate the terminal growth and branching of axons of NGF-dependent neurons in certain target tiisues.

Here we report that growth differentiation factor-5 (GDF5), a widely expressed member of the bone morphogenetic protein/growth differentiation factor (BMP/GDF) family which constitutes a collection of closely related proteins within the TGFβ superfamily [7], is a novel regulator of sympathetic axon growth, acting during the stage in development when the axon terminals of NGF-dependent sympathetic neurons of the superior cervical ganglion (SCG) neurons are growing and branching extensively in their targets in vivo. GDF5 has extensively characterized roles in chondrogenesis, joint formation, skeletal, tendon and ligament morphogenesis [8–10]. Several reports have also implicated GDF5 in neuronal development. GDF5 enhances the survival of embryonic midbrain dopaminergic neurons and promotes neurite outgrowth from these neurons in vitro [11, 12], it promotes the growth and elaboration of pyramidal cell dendrites in the embryonic hippocampus both in vitro and in vivo [13] and has a minor survival-promoting action on embryonic chicken dorsal root ganglion (DRG) neurons in vitro [14]. Here we report and characterize novel, physiologically relevant functions for GDF5 in sympathetic neuron development. We show that GDF5 promotes the growth and branching of sympathetic axons during a narrow window of postnatal development and functions as a novel target-derived regulator of sympathetic innervation in vivo that makes a distinctive regional contribution to the establishment of sympathetic innervation.

## Results

### GDF5 enhances neurite growth and branching from neonatal SCG neurons

**Fig. 1:**
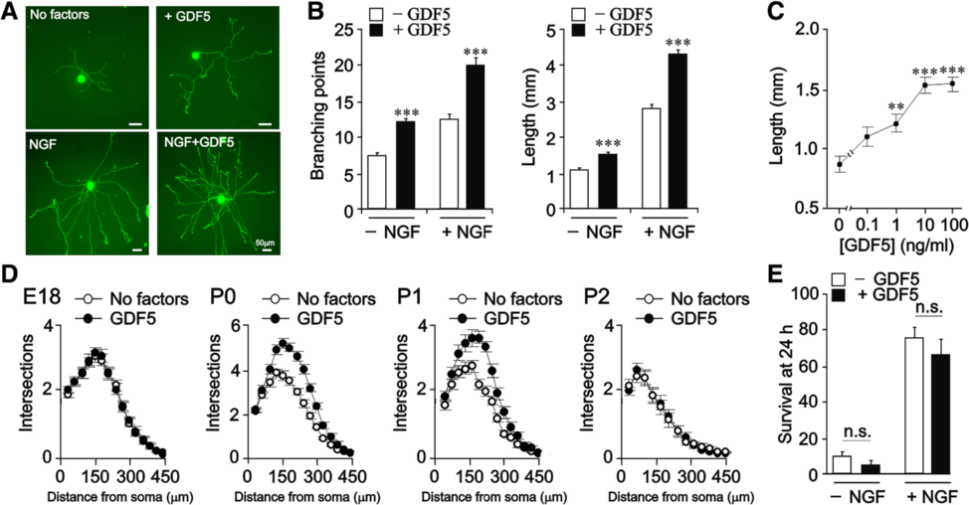
GDF5 promotes neurite growth from cultured neonatal SCG neurons. **a** Photomicrographs of representative P0 SCG neurons cultured for 24 h with or without 10 ng/ml GDF5 and/or 10 ng/ml NGF. **b** Branch point number and total length of neurite arbors of P0 SCG neurons cultured for 24 h with or without 10 ng/ml GDF5 and/or 10 ng/ml NGF. **c** Neurite arbor lengths of P0 SCG neurons cultured for 24 h with different concentrations of GDF5. **d** Sholl analysis of the arbors of E18, P0, P1 and P2 SCG neurons cultured for 24 h with and without 10 ng/ml GDF5. Cultures without NGF received 50 μM Boc^_^D^_^FMK to prevent apoptosis. Mean ± SEM of data from at least 150 neurons in each condition from 3 independent experiments are shown (** *P* < 0.01 and *** *P* < 0.001, statistical comparison with control, one-way ANOVA with Fisher’s post hoc). **e** Percentage survival of P0 SCG neurons cultured for 24 h in the absence of Boc^_^D^_^FMK with or without 10 ng/ml GDF5 in the absence or presence of 10 ng/ml NGF (mean ± SEM of the results of 3 independent experiments; n.s. = not significant)

GDF5 increased the size of the neurite arbors of newborn mouse (P0) SCG neurons cultured at low density in defined medium. It enhanced neurite arbor size of neurons cultured with saturating concentrations of NGF and promoted neurite growth from neurons cultured without NGF in medium containing the broad-spectrum caspase inhibitor Boc^_^D^_^FMK to prevent apoptosis of the neurons in the absence of NGF (Fig. 1a). Analysis of the neurite arbors of large numbers of neurons showed that GDF5 promoted highly significant increases in overall neurite length and the number of branch points in both the presence and absence of NGF (Fig. 1b). Dose response analysis revealed that the neurite growth-promoting effect of GDF5 reached saturation at 10 ng/ml (Fig. 1c).

Analysis of the influence of GDF5 on SCG neurons in cultures established over a range of ages revealed that the neurite growth enhancing effect of GDF5 was restricted to a narrow developmental window in the immediate postnatal period. Sholl analysis, which provides a graphic illustration of neurite length and branching with distance from the cell body, showed that the effect of GDF5 on neurite growth and branching was evident at P0 and P1 but not before or after this period (Fig. 1d). This contrasts with the neurite growth promoting effect of NGF, which is evident from the time the earliest sympathetic axons reach their targets to well after the phase of programmed cell death [15–17]. In marked contrast to NGF, which promotes the survival of SCG neurons in addition to promoting neurite growth, GDF5 did not promote the survival of SCG neurons on its own and did not increase the number of neurons surviving with NGF (Fig. 1e). These results show that GDF5 enhances neurite growth from sympathetic neurons independently of NGF without affecting neuronal survival.

Because the neurites in short^_^term SCG cultures are exclusively axons [18], confirmed by absence of dendrite markers in SCG neurons cultured with or without GDF5 for 24 h (not shown), our findings suggest that GDF5 promotes axon growth and branching from SCG neurons independently of NGF without affecting neuronal survival over a narrow developmental window during the period when sympathetic axon terminals are growing and branching within their targets.

### GDF5 acts locally on axon terminals to promote axon growth

**Fig. 2:**
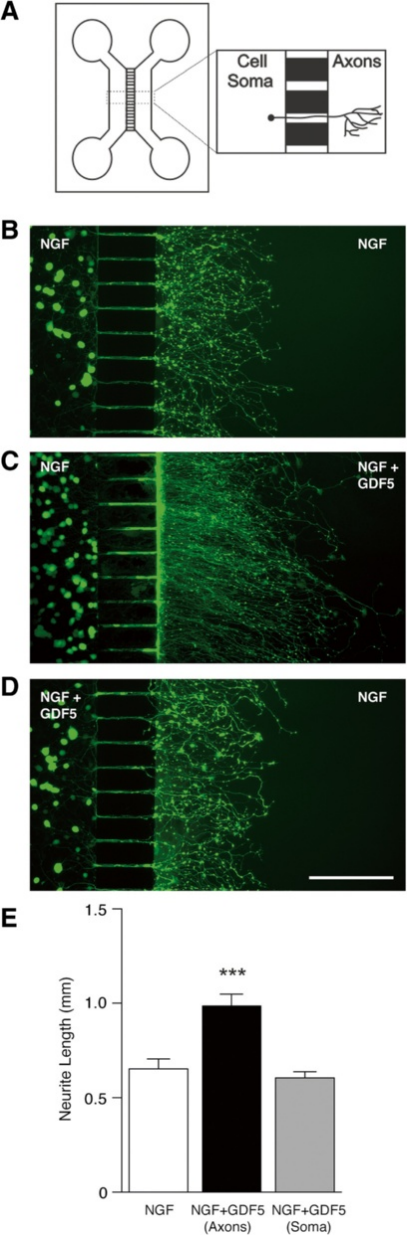
GDF5 acts locally on axon terminals to promote axon growth. **a** Schematic illustration of the two-chamber microfluidic device. The inset shows a neuron with its cell body in the soma compartment on the left extending its axon arbor through a microchannel into the axon compartment on the right. **b**-**d** Photomicrographs of representative P0 SCG neurons cultured in defined medium for 24 h with 10 ng/ml NGF in both compartments (**b**), NGF in both compartments plus 10 ng/ml GDF5 in the axonal compartment (**c**) and NGF in both compartments plus 10 ng/ml GDF5 in the soma compartment (**d**). Scale bar = 200 μm. **e** Bar chart of axon length measurements in the axon compartment (*** *P* < 0.0001, statistical comparison with NGF alone, ANOVA)

To determine if, like NGF, GDF5 is capable of acting locally on axons to promote their growth, we cultured these neurons in microfluidic devices in which the cell bodies and axon terminals are grown in separate compartments (Fig. 2a). P0 SCG neurons were seeded into one compartment of a two-compartment device that contained NGF in both compartments. After 24 h incubation, maximal axon length in the axon compartment was quantified. Whereas supplementation of the axon compartment by GDF5 caused a highly significant increase in axon length compared to NGF alone, supplementation of the soma compartment by GDF5 did not enhance axon length compared to NGF alone (Fig. 2b-e). It was also clearly evident that addition of GDF5 to the axon compartment, but not the soma compartment, increased neurite density in the axon compartment (Fig. 2b-d). These data show that GDF5 acting locally on sympathetic axons, but not on neuronal soma, significantly increases axon growth

### Expression of GDF5 receptors on developing SCG neurons

Members of the BMP/GDF family mediate their actions via a heterodimeric complex of type I and type II serine-threonine kinase receptors [19]. Among type I receptors, GDF5 binds most efficiently to BMPR1B expressed alone on cell lines but binds weakly to BMPR1A when co-expressed with ACVR2A (ActRII). Receptor reconstitution experiments in Mv1Lu epithelial cells have shown that GDF5 is able to transduce a signal via BMPR1B in combination with either ACVR2A or BMPR2 (BMPRII), but is also able to signal somewhat less effectively via BMPR1A in combination with ACVR2A [20].

**Fig. 3:**
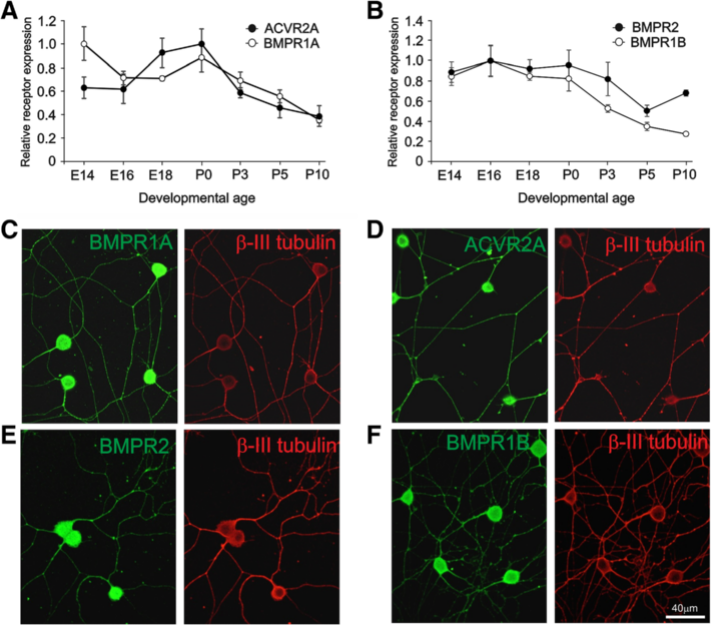
Expression of GDF5 receptors. **a**, **b** Expression of transcripts encoding GDF5 receptors in the SCG at stages from E14 to P10 relative to the geometric mean of *Gapdh*, *Sdha* and *Hprt1* reference mRNAs (mean ± SEM, n = 3 per age). **a** Relative levels of *Bmpr1a* and *Acvr2a* mRNAs normalised to the peak of expression at E14 and P0, respectively. **b** Relative levels of *Bmpr1b* and *Bmpr2* mRNAs normalised to the peak of expression at E16. **c to f** Photomicrographs of representative P0 SCG neurons cultured for 24 h with NGF and double labelled for β-III tubulin and either BMPR1A (**c**), ACVR2A (**d**), BMPR2 (**e**) or BMPR1B (**f**). Scale bar = 40 μm

Transcripts encoding BMPR1B, BMPR2, BMPR1A and ACVR2A were expressed in the developing SCG from E14 to at least P10, which spans the period over which SCG axons first reach their target tissues and sympathetic innervation is established. The expression profiles for *Bmpr1a* mRNA and *Acvr2a* mRNA (Fig. 3a) and *Bmpr1b* mRNA and *Bmpr2* mRNA (Fig. 3b) showed an overall small decrease from E14 to P10.

Immunocytochemistry revealed that antibodies to each receptor stained the neurites of virtually all P0 SCG neurons in culture (Fig. 3c to f). Cultures incubated with secondary antibody alone exhibited no background immunofluorescence (not shown). These studies suggest that receptor complexes containing BMPR1B and/or BMPR1A are available for mediating the neurite growth-promoting effects of GDF5.

### GDF5 promotes retrograde canonical Smad signalling along SCG axons

**Fig. 4:**
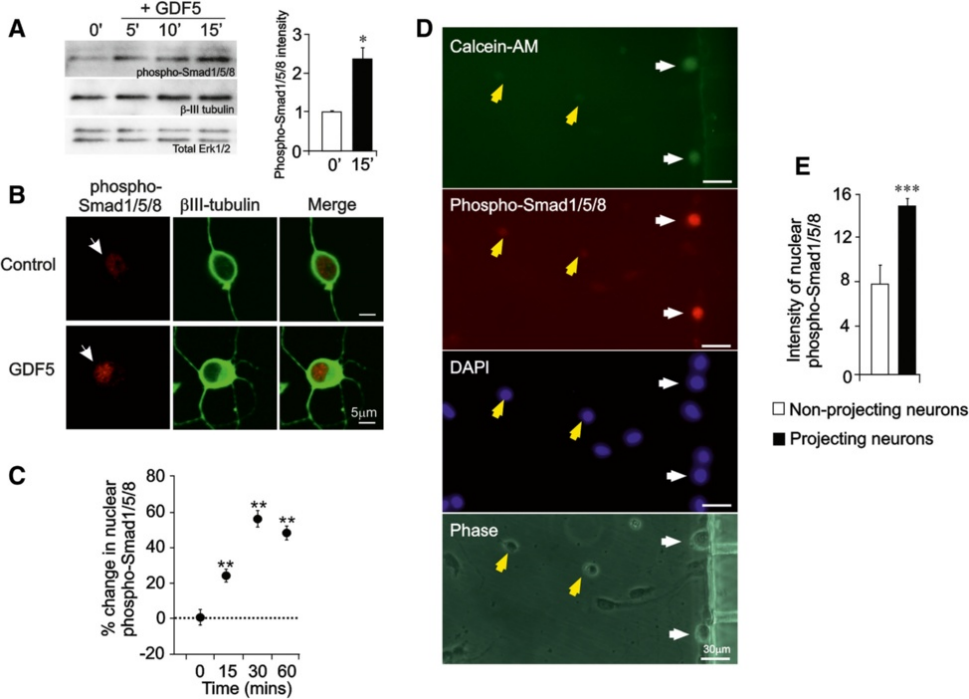
GDF5 promotes retrograde canonical Smad signalling along SCG axons. **a** Representative Western blot for phospho-Smad1/5/8 in P0 SCG neurons treated with GDF5 for 5, 10, and 15 min or untreated (0′) 12 h after plating using βIII tubulin and total ERK1/2 as loading standards. The bar chart plots the relative levels of phospho-Smad1/5/8 from densitometry of multiple blots at the 0 and 15 min time points (mean ± SEM). **b** Representative P0 SCG neurons immunolabeled for phospho-Smad-1/5/8 and β-III tubulin after either 30 min treatment with GDF5 or untreated (Control) 12 h after plating. **c** Percentage increase in nuclear phospho-Smad-1/5/8 immunolabeling in P0 SCG neurons treated with GDF5 for the indicated times relative to untreated control levels. **d** Representative P0 SCG neurons immunolabeled for phospho-Smad-1/5/8 after 60 min treatment of their axons with 10 ng/ml GDF5 in compartment cultures 12 h after plating. Addition of calcein-AM to the axon compartment was used to identify neurons that had projected axons into the axon compartment (white arrows), whereas unlabelled cells (yellow arrows) had not projected axons into the axon compartment. DAPI labelling indicates all cell nuclei in the field and the phase contrast image shows the location of the compartment barrier with two of its microchannels. Neurons whose axons had been exposed to GDF5 had a clear increase in nuclear accumulation of phospho-Smad proteins. Scale bar = 30 μm. **e** Relative intensity of nuclear phospho-Smad immunofluorescence in neurons with axons that had or had not projected into the axon compartment (mean ± SEM, *P < 0.05, ** *P* < 0.01, *** *P* < 0.001, statistical comparison with control, one-way ANOVA with Fisher’s post hoc)

In common with other members of the BMP/GDF family, GDF5 activates canonical Smad 1/5/8 signaling [21], which is required for GDF5 responsiveness in neurons [13]. To ascertain whether GDF5 could initiate retrograde Smad signaling to the nucleus, we first confirmed that GDF5 is able to activate Smads 1/5/8 in SCG neurons. Western blot analysis showed that GDF5 increased Smad 1/5/8 phosphorylation in P0 SCG neurons as early as 5 minutes after GDF5 treatment (Fig. 4a). Immunocytochemistry using an antibody that recognizes phospho-Smad1/5/8 revealed that GDF5 promoted rapid nuclear accumulation of phospho-Smad1/5/8 in dissociated cultures of SCG neurons (Fig. 4b and c).

To assess retrograde Smad signaling, P0 SCG neurons were incubated for 12 h in compartment cultures containing NGF in both compartments before GDF5 was added to the axon compartment. After an hour, calcein-AM was added to the axon compartment to identify neurons with axons projecting into this compartment. The cultures were then fixed and stained for nuclear phospho-Smad-1/5/8. GDF5 treatment of sympathetic axons resulted in a highly significant increase in nuclear phospho-Smad1/5 immunoreactivity in neurons that projected axons into the axon compartment, but not in those neurons whose axons did not cross into this compartment (Fig. 4d and e). This demonstrates that GDF5 acting on sympathetic axons induced retrograde canonical Smad signaling along these axons, leading to nuclear accumulation of phospho-Smad proteins.

**Fig. 5:**
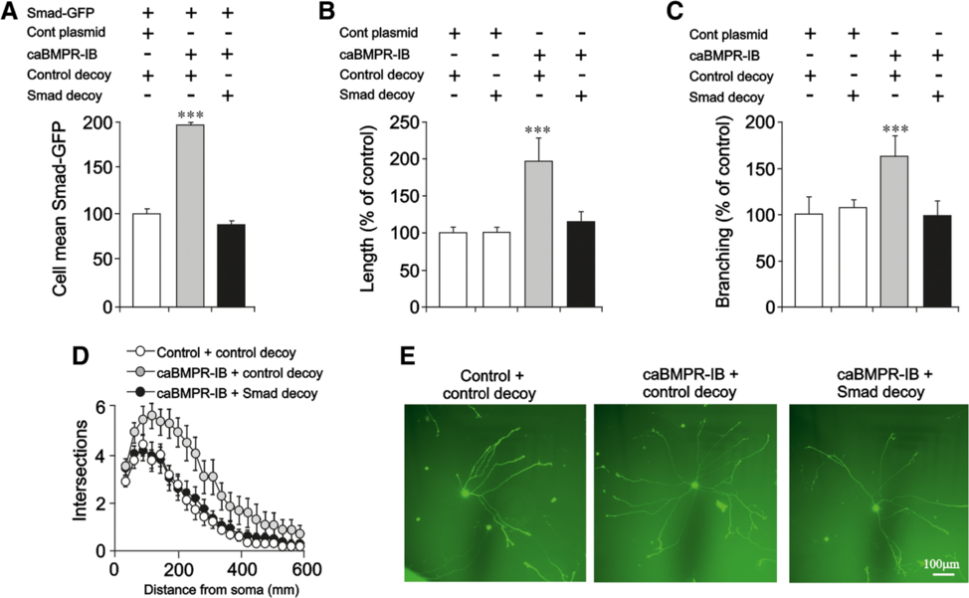
Canonical Smad signalling promotes neurite growth in SCG neurons. **a** Smad-dependent transcriptional activity in P0 SCG neurons 24 h after transfection with the Smad-GFP reporter plus either the caBMPR-IB plasmid or pcDNA3.1 control plasmid and either Smad decoy oligonucleotides or scrambled control decoy oligonucleotides. **b** Length, **c** branch number and **d** Sholl analysis of the neurite arbors of P0 SCG neurons 24 h after transfection with the indicated plasmids and oligonucleotides. For clarity, the Sholl plot includes only one control plasmid condition. **e** Photomicrographs of representative P0 SCG neurons 24 h after transfection with the plasmids indicated. Mean ± SEM of data from at least 150 neurons in each condition from 3 independent experiments are shown (*** *P* < 0.001, statistical comparison with control, one-way ANOVA with Fisher’s post hoc)

To test directly the importance of GDF5 receptor-dependent Smad activation and Smad-dependent gene transcription on neurite growth, we co-transfected P0 SCG neurons with a reporter construct in which GFP is under the control of Smad binding elements together with either a pcDNA plasmid that expresses a constitutively active BMPR-IB (caBMPR-IB) [22] or an empty pcDNA control plasmid. Quantification of the reporter signal 24 h after transfection revealed that the caBMPR-IB promoted a highly significant increase in Smad-dependent gene transcription that was completely prevented by a co-transfected decoy double-stranded DNA-oligonucleotide that contains the Smad consensus binding sequence but not by a control oligonucleotide with a scrambled sequence (Fig. 5a). The caBMPR-IB also promoted highly significant increases in neurite length (Fig. 5b), branch point number (Fig. 5c) and overall neurite arbor size and complexity (Fig. 5d and e) that were completely prevented by the Smad oligonucleotide decoy but not by the control oligonucleotide. There were no significant differences in the length and branching of the neurite arbors of neurons transfected with the control plasmid plus either decoy DNA or scrambled DNA (Fig. 5b-c).

### GDF5 plays a distinctive role in establishing sympathetic innervation in vivo

To ascertain whether the increase in sympathetic axon growth and branching brought about by GDF5 in vitro contributes to the establishment of sympathetic innervation in vivo, we used tyrosine hydroxylase immunofluorescence to identify sympathetic fibers and quantify sympathetic innervation density in wild type mice and mice that are either heterozygous or homozygous for the *Gdf5*^bp^ null mutation, a frame-shift mutation in the *Gdf5* gene [23]. For this analysis, we studied the submandibular salivary gland, trachea and iris. While the terminal growth and branching of sympathetic axons in the submandibular salivary gland is dependent on NGF, the terminal ramification of sympathetic axons in the trachea occurs independently of NGF [5]. Our analysis was carried out at P10, which is after the period of development when GDF5 enhances neurite growth in vitro and is at a stage in vivo when the sympathetic innervation of these tissues has become well established.

**Fig. 6:**
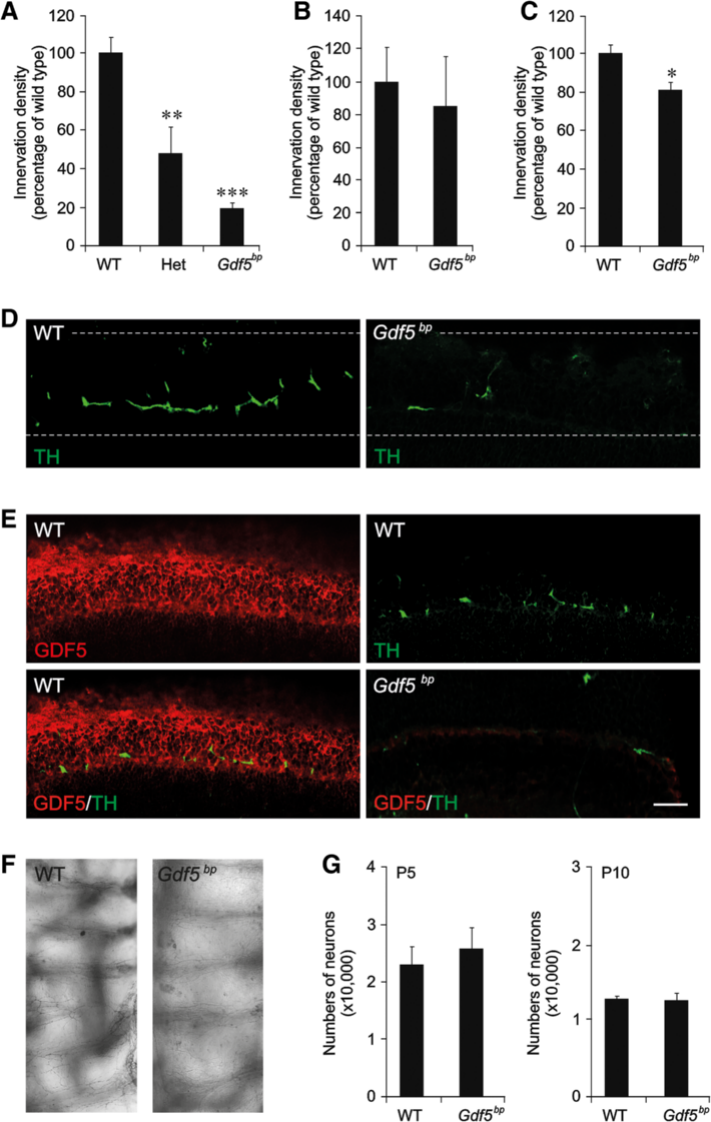
Selective sympathetic innervation deficits in mice possessing the *Gdf5* ^*bp*^ mutation. **a**-**c** Relative innervation density assessed by quantification of tyrosine hydroxylase-positive sympathetic fibres at P10 in the irides (**a**), submandibular glands (**b**) and trachea (**c**) of wild type mice (WT) and mice that are heterozygous (Het) or homozygous (*Gdf5* ^*bp*^) for the *Gdf5* ^*bp*^ null mutation. The level of tyrosine hydroxylase staining is normalised to 100 for the tissue of wild type mice (mean ± SEM, *P < 0.01, **P < 0.00001, ***P < 0.0000001, statistical comparison with wild type, one-way ANOVA with Tukey HSD post hoc, n = 10 animals per genotype for the iris, n = 5 animal per genotype for the submandibular gland and the trachea). **d** Representative sections of the irides of P10 wild type and *Gdf5* ^*bp*^ mice stained for tyrosine hydroxylase. The boundaries of the iris are indicated by the dashed lines. **e** Representative sections of the iris of a P10 wild type and *Gdf5* ^*bp*^ mice double stained for GDF5 and tyrosine hydroxylase. Scale bar = 100 μm. **f** Representative whole mounts P10 wild type and *Gdf5* ^*bp*^ mice stained for tyrosine hydroxylase. Scale bar = 100 μm. **g** Numbers of neurons in the SCG of P5 and P10 wild type and *Gdf5* ^*bp*^ mice (n = 3 per genotype)

Quantification of tyrosine hydroxylase immunofluorescence in tissue sections revealed marked, highly significant reductions in the iris of heterozygous and homozygous mice compared with wild type mice (Fig. 6a). The level of tyrosine hydroxylase immunoreactivity in the irides of heterozygous mice was intermediate between that of wild type and homozygous mice, indicative of a gene dosage effect. There was no significant reduction in the level of tyrosine hydroxylase immunoreactivity in the submandibular salivary gland of *Gdf5*^bp^ mice compared with wild type mice (Fig. 6b). Because there are fewer sympathetic fibers in the trachea, we assessed innervation density by quantifying the density of tyrosine-hydroxylase-positive fibers in cleared whole-mount tissue preparations. This analysis revealed a statistically significant reduction in tyrosine hydroxylase-positive nerve fiber density in *Gdf5*^bp^ mice compared with wild type mice (Fig. 6c). Figure 6d illustrates representative tyrosine hydroxylase-immunolabeled sections through the irides of wild type mice and *Gdf5*^*bp*^ mice, illustrating the clear decrease in sympathetic innervation density in the latter. To determine the site of GDF5 expression and to ascertain its relation to sympathetic fibers in the iris, we double labeled sections of the iris for tyrosine hydroxylase and GDF5. This showed prominent GDF5 immunoreactivity throughout the stroma and tyrosine hydroxylase-positive fibers ramifying within the stroma (Fig. 6e). GDF5 immunoreactivity was not observed in the irides of *Gdf5*^*bp*^ mice, demonstrating the specificity of the anti-GDF5 antibody used (Fig. 6e). These observations show that GDF5 is expressed by cells in the target field. Figure 6f illustrates representative tyrosine hydroxylase-immunolabeled whole mounts of the trachea of wild type mice and *Gdf5*^*bp*^ mice.

Neuron counts in the SCG at P5 and P10 revealed no significant differences between wild type mice and *Gdf5*^*bp*^ mice at each age (Fig. 6g), suggesting that SCG neurons are not dependent on GDF5 for survival when sympathetic innervation is being established during the postnatal period. Taken together, these findings suggest that GDF5 plays a tissue-selective role in establishing sympathetic innervation in vivo without affecting SCG neuron number.

## Discussion

We have discovered and characterized novel and distinctive functions for GDF5 in the developing peripheral nervous system and show that these differ in several respects from the potential roles of GDF5 reported for other neurons. First, whereas GDF5 promotes the growth and branching of axons from sympathetic neurons, GDF5 promotes the growth and elaboration of dendrites but not axons from hippocampal pyramidal cells [13]. Second, whereas GDF5 neither promotes the survival of sympathetic neurons alone nor enhances the survival of these neurons in combination with NGF, GDF5 has a minor survival-promoting action on DRG neurons alone and enhances the survival-promoting action of NGF and NT3 in vitro [14]. GDF5 also promotes the survival of midbrain dopaminergic neurons in vitro [12] and is neuroprotective for these neurons exposed to toxic insults both in vitro [11, 24] and in vivo [25]. Third, whereas the effects of GDF5 on sympathetic neurons are restricted to a very narrow postnatal window of development, midbrain dopaminergic neurons remain responsive to GDF5 from embryonic stages into the adult [11, 12, 25]. Our current study together with previous work documents the multiple distinctive actions of GDF5 in the developing and mature nervous system.

In addition to GDF5, several other factors have been shown to enhance sympathetic axon growth and branching over restricted windows of development during the stage when sympathetic axons are ramifying extensively in their targets, without affecting neuronal survival. GDF5 and these other factors do not have redundant functions, but act by different mechanisms and have distinctive roles in establishing sympathetic innervation in vivo.

Both TNFR1 [26], acting via a TNF reverse signaling mechanism, and GDF5 act in a target-derived manner. TNFR1 and GDF5 are expressed in target tissues and act on sympathetic axon terminals to promote growth and branching. However, whereas studies of *Gdf5*^*bp*^ mice reveal a distinctive regional requirement for GDF5 in vivo in the establishment of sympathetic innervation, multiple tissues display defective sympathetic innervation in both *Tnfr1*−/− and *Tnf*−/− mice [25]. It is not clear why GDF5 plays a major role in establishing sympathetic innervation in the iris and to a lesser extent in the trachea, but not in the submandibular gland. Like TNFR1, GDF5 is widely expressed in multiple tissues, and the level of GDF5 is no higher in the iris than in the submandibular gland (data not shown). For this reason, the marked innervation defect in iris of *Gdf5*^bp^ mice cannot be explained simply by regional differences in GDF5 expression and access of sympathetic axons to GDF5. Whether a subset of SCG neurons that innervate the iris and trachea respond to GDF5 in vivo, either because they are pre-specified to respond or because target-derived factors regulate GDF5 responsiveness, is an intriguing issue for future work. The trachea is the one tissue of the many analyzed whose sympathetic innervation is completely independent of NGF [5]. The small, though significant, decrease in the sympathetic innervation of the trachea of *Gdf5*^bp^ mice shows that GDF5 makes a significant contribution to establishing tracheal sympathetic innervation. The other factor or factors required for promoting the ramification of sympathetic fibres in this tissue remain elusive.

Like GDF5, GITR signaling and CD40 signaling enhance sympathetic axon growth during the immediate postnatal period without affecting neuronal survival and play roles in establishing sympathetic innervation in vivo [27, 28]. However, in contrast to the target-derived mode of action of GDF5, GITR signaling and CD40 signaling act by an autocrine mechanism. Also, in contrast to GDF5, which promotes sympathetic axon growth alone in the absence of NGF, stimulating these autocrine signaling loops in the absence of NGF does not promote sympathetic axon growth. Rather, these autocrine signaling loops enhance the axon growth-promoting actions of NGF [27, 28]. Despite modulating NGF responsiveness, *Cd40*−/− mice, like *Gdf5*^*bp*^ mice, display regional deficits in sympathetic innervation density [28]. This is because NGF negatively regulates the expression of both CD40 and its autocrine signaling partner CD40L, with the result that these proteins are only expressed at functionally relevant levels in low NGF-expressing tissues, which are those that are selectively hypo-innervated in *Cd40*−/− mice [28].

HGF [29] and Wnt5a [30] are another two factors that enhance sympathetic axon growth by an autocrine mechanism during the stage of development when sympathetic axons are ramifying in their final targets. While this activity has only been reported for HGF in vitro, the importance of Wnt5a has been demonstrated in vivo in mice in which *Wnt5a* is conditionally inactivated in sympathetic neurons. In these mice, sympathetic fibres reach their final targets, but display greatly reduced growth and terminal arborization in multiple tissues, which contrasts with the regional deficit observed in *Gdf5*^*bp*^ mice.

Most SCG neurons are generated between E11.5 and birth [31, 32], the earliest sympathetic fibres reach SCG targets at E13 [33] and the density of sympathetic innervation of many tissues clearly increases between the late fetal and early postnatal period [5]. Thus, the brief window over which GDF5 enhances axon growth from SCG neurons appears to occur shortly after sympathetic axons have arrived at their target tissues and are ramifying within and refining connections in these tissues. GDF5 binds efficiently to BMPR1B and transduces a signal via BMPR1B in combination with either BMPR2 or ACVR2A. However, GDF5 also binds BMPR1A weakly when co-expressed with ACVR2A and transduces a signal in cell lines co-expressing BMPR1A and ACVR2A [20]. We show that SCG neurons express BMPR1A and ACVR2A as well as BMPR1B and BMPR2 during this brief window of GDF5 responsiveness. However, we find that transcripts for all receptors are expressed at similar levels in the SCG for extended periods before and after the window of GDF5 responsiveness. This suggests that the timing of GDF5 responsiveness is unlikely to be due to changes in receptor expression. This contrasts with enhanced SCG axon growth brought about by activating TNF reverse signaling and by activating the extracellular Ca^2+^ sensitive receptor (CaSR). The developmental window of responsiveness of SCG neurons to TNFR1 and to elevated extracellular Ca^2+^ coincides with peaks in TNF [26] and CaSR expression [34]. Moreover, experimental elevation of CaSR expression after the normal peak of expression re-confers responsiveness to elevated extracellular Ca^2+^ [34]. Our current findings raise the possibility that the timing of GDF5 responsiveness might be due to developmental changes in GDF5 local signaling, retrograde GDF5 signaling or gene regulation.

GDF5 rapidly activates canonical Smad 1/5/8 signaling in hippocampal pyramidal cells and rat midbrain dopaminergic neurons, and this in turn is required for GDF5-promoted neurite growth and dendrite growth [13, 35]. Here we show that GDF5 also rapidly activates Smad 1/5/8 signaling in sympathetic neurons, and demonstrate using compartment cultures that treating sympathetic axons with GDF5 results in the rapid appearance of phospho-Smad immunoreactivity in the nucleus. This suggests that GDF5 induces retrograde Smad signaling. BMP4 also initiates retrograde Smad signaling along trigeminal sensory axons, and this plays a role in conveying spatial patterning information from the periphery in the developing trigeminal system [36]. Moreover, we show that decoy DNA encoding the Smad consensus binding sequence inhibits the enhanced axon growth associated with GDF5 receptor-dependent Smad activation, suggesting that Smad-dependent gene transcription enhances axon growth in developing sympathetic neurons. While these data suggest that retrograde Smad signaling to the nucleus is important for GDF5-promoted axon growth, compartment culture experiments show that GDF5 added to the axon compartment enhances axon growth, whereas GDF5 added to the soma compartment does not. This observation raises the possibility that retrograde Smad signaling alone is insufficient for GDF5-enhanced axon growth, but that local signaling is also required. This has parallels with other target-derived neurotrophic factors. For example, whereas retrograde NGF/TrkA signaling to the nucleus is required for NGF-promoted neuron survival, local TrkA-dependent signaling events in at the growth cone are required for axon growth promoted by NGF and NT3 [3, 37]. In future work it will be interesting to dissect the local signaling events required for GDF5-promoted axon growth.

Our current work demonstrates that GDF5 is one of a growing number of factors that promote, by a diversity of mechanisms, sympathetic axon growth and branching and have distinctive roles in establishing sympathetic innervation in vivo without affecting neuronal survival. In addition, factors expressed in target tissues such as RANKL inhibit sympathetic axon growth and branching, at least in vitro, without affecting neuronal survival [38]. In contrast, target-derived NGF performs the key dual roles of sustaining sympathetic neuron survival and promoting the growth and ramification of sympathetic axons in target tissues in vivo. Indeed, there is overwhelming evidence that the limited availability of NGF in different tissues governs the number of neurons that survive the phase of naturally occurring death to innervate these tissues [1]. Given this dual function, why are additional factors that regulate axon growth but not survival required? The most likely reason is that these additional factors permit regional adjustments in innervation density by the limited number of NGF-supported neurons that innervate particular tissues.

## Conclusions

We have discovered and characterized novel and distinctive functions for GDF5 in the developing nervous system. GDF5 promotes the growth and branching of sympathetic axons independently of NGF without affecting sympathetic neuron survival during a brief window of postnatal development when sympathetic axons are ramifying within their targets. GDF5 is expressed in sympathetic targets, acts directly on sympathetic axons and initiates retrograde Smad signaling along these axons to the nucleus. In vivo, there is a distinctive regional requirement for GDF5 in the establishment of sympathetic innervation, being required in the iris, not in the submandibular gland and making a small, significant contribution to the trachea, whose innervation is independent of NGF. These findings extend our understanding of the physiology of GDF5 and provide a deeper understanding of the mechanisms that pattern sympathetic innervation during development.

## Methods

### Mice

C57BL6/J and brachypod (*Gdf5*^bp^) mice were obtained from the Jackson Laboratory (Bar Harbor, Maine, USA). While *Gdf5*^bp^ mice are recognized by their short limb phenotype, wild type and heterozygous mice are phenotypically indistinguishable and cannot be genotyped by standard PCR-based methods. To generate litters consisting of mice that are homozygous and heterozygous for the *Gdf5*^bp^ mutation, a female *Gdf*5^bp^ mouse was crossed with a male mouse that is heterozygous for the *Gdf5*^bp^ mutation. Heterozygous male mice were identified as phenotypically normal mice that produced litters comprising phenotypically normal mice and *Gdf5*^bp^ mice when crossed with *Gdf5*^bp^ females. Age-matched wild type mice were obtained by crossing C57BL6/J mice. All other studies were carried out on tissues obtained from CD-1 mice.

### Real-time PCR quantification of mRNA levels

SCG were harvested from embryonic and postnatal mice. Because whole ganglia contain both neurons and satellite cells, the RNA used for these studies is derived from both cell types. Total RNA was isolated with the RNeasy Mini extraction kit (Qiagen, Germany) and was reverse transcribed with AffinityScript RT (Agilent). Reverse transcription (RT) reactions were amplified using the Brilliant III ultra-fast QPCR master mix (Agilent). QPCR assays for *Bmpr1a*, *BmprIb*, *Acvr2a*, *Bmpr2* and the reference genes, *Gapdh*, *Sdha* and *Hprt1* used dual-labeled probes to detect PCR products. The forward and reverse primers were as follows: *Gapdh*, 5′-gagaaacctgccaagtatg-3′ and 5′-gggttgctgttgaagtc-3′; *Sdha*, 5′-ggaacactccaaaaacag-3′ and 5′-ccacagcatcaaattcat-3′; *Hprt1*, 5′-ttaagcagtacagccccaaaatg-3′ and 5′-aagtctggcctgtatccaacac-3′; *Bmpr1a*, 5′-tacgcaggacaatagaat-3′ and 5′-aactatacagacagccat-3′; *Bmpr1b*, 5′-agtgtaataaagacctcca-3′ and 5′-aactacagacagtcacag-3′; *Bmpr2*, 5′-actagaggactggcttat-3′ and 5′-ccaaagtcactgataacac-3′; *Acvr2a*, 5′-cgccgtctttcttatctc-3′ and 5′-tgtcgccgtttatcttta-3′. Dual labeled probes were: *Gapdh*, 5′- FAM-agacaacctggtcctcagtgt-BHQ1-3′; *Sdha*, 5′-FAM-cctgcggctttcacttctct-BHQ1-3′; *Hprt1*, 5′-FAM-tcgagaggtccttttcaccagcaag-BHQ1-3′; *Bmpr1a*, 5′- FAM-tgagcacaaccagccatcg-BHQ1-3′; *Bmpr1b*, 5′-FAM-ccactctgcctcctctcaag-BHQ1-3′; *Bmpr2*, 5′-FAM-cacagaattaccacgaggaga-BHQ1-3′; *Acvr2a*, 5′-FAM-tgctcttcaggtgctatacttggc-BHQ1-3′. The PCR was performed (Mx3000P, Stratagene) for 40 cycles of 95 °C for 10 sec, and 60 °C for 30 sec. Standard curves were generated for every PCR run with each primer/probe set using serial five-fold dilutions of adult mouse brain RT RNA (Zyagen). *Bmpr1a*, *Bmpr1b*, *Bmpr2* and *Acvr2a* mRNAs were expressed relative to the geometric mean of the reference mRNAs.

### Neuron cultures

Dissected SCG were trypsinized and the neurons were plated at very low density (~200 neurons per dish) in poly-ornithine/laminin-coated 35 mm tissue culture dishes (Greiner, Germany) in serum-free Hams F14 medium supplemented with 0.25 % Albumax I (Life Technologies, Paisley, UK) [39]. NGF (Calbiochem, UK), GDF5 (Biopharm, Germany) and the broad^_^spectrum caspase inhibitor Boc^_^D^_^FMK (Calbiochem, UK) were added as indicated.

Neuronal survival was estimated by counting the number of attached neurons within a 12 × 12 mm grid in the centre of each dish 2 h after plating and again after 24 h, and expressing the 24-h count as a percentage of the 2-h count [39]. Analysis of the size and complexity of neurite arbors was carried out 24 h after plating. The neurite arbors were labeled by incubating the neurons with the fluorescent vital dye calcein-AM (1:1000, Invitrogen, Paisley, UK). Images of neurite arbors were acquired by fluorescence microscopy and analyzed to obtain total neurite length, number of branch points and Sholl profiles [40].

For compartment cultures, the neurons were seeded in one compartment of a two compartment microfluidic device (Xona Microfludics, CA, USA) and the medium in both compartments was supplemented with 10 ng/ml NGF to promote the growth of axons in both compartments. 10 ng/ml GDF5 was added either to the axon compartment or the soma compartment at plating, and axon growth was assessed 24 h later following addition of the fluorescent vital dye Calcein-AM (Invitrogen) to the axonal compartment to label axons in this compartment. ImageJ was used to measure the lengths of the 10 longest axons per random field (distances to the 10 furthest growth cones from the compartment barrier) to obtain a mean measurement for each field. Means of these measurements were obtained from 7 separate random fields along the microfluidic barrier of each compartment culture.

For the retrograde signaling experiments, the neurons were incubated with 10 ng/ml NGF in both compartments for 12 h before 10 ng/ml GDF5 was added to the axon compartment, followed by addition of calcein-AM to identify which neurons had projected axons into the axonal compartment. Cultures where immediately fixed and stained for phospho-Smad, as described below.

### Neuron transfection and reporter assays

SCG neurons were transfected as previously described [41] with the GFP-based Cignal Smad Reporter (SABiosciences, UK) and either an empty control plasmid or the caBMPR-IB plasmid together with either a Smad decoy (double stranded DNA oligonucleotide, 5′-gtacattgtcagtctagacataact-3′) prepared as described previously or a scrambled control decoy oligonucleotide (double stranded DNA oligonucleotide, 5′-atcataatttggaactgtagtccg-3′). Reporter activity is expressed as the mean fluorescence intensity of individual cells as previously described [41].

### Immunocytochemistry

Cultures were fixed in 4 % paraformaldehyde at room temperature for 10 min, washed with phosphate buffered saline (PBS) and blocked with 5 % bovine serum albumen (BSA) and 0.1 % TritronX-100 in PBS for 1 h at room temperature. The cells were incubated overnight with primary antibody in 1 % BSA at 4 °C. The following primary antibodies were used: rabbit polyclonal anti-BMPR1A (Abcam, Cambridge, UK, 1/200, catalogue number ab38560), rabbit polyclonal anti-BMPR1B (Abcam, Cambridge, UK, 1/200, catalogue number ab175385), rabbit polyclonal anti-BMPR2 (Abcam, Cambridge, UK, 1/200, catalogue number ab124463), rabbit polyclonal anti-ACVR2A (Abcam, Cambridge, UK, 1/200, catalogue number ab96793), rabbit polyclonal anti-phospho-Smad1/5 (Ser463/465) (41D10) (Cell Signaling, Danvers, MA, USA, 1:100, catalogue number 9516) and β-III tubulin (Abcam, Cambridge, UK, 1:500, ab41489). After washing, the cultures were incubated with an Alexa-Fluor-labeled secondary antibody (1:500 Alexa Fluor 488 anti-rabbit, Life Technologies, UK, catalogue number A-1108 and 1:500 Alexa Fluor 594 anti-chicken, Abcam, Cambrdige, UK, catalogue number ab150172) for 1 h and counterstained with DAPI (Chemicon) where indicated. Quantification of nuclear staining of phospho-Smad1/5/8 was carried out as described [42].

### Immunohistochemistry

The iris and submandibular salivary gland of wild type and mice that are heterozygous or homozygous for the GDF-5^bp^ mutation were fixed in 4 % paraformaldehyde and 0.1 % TritonX-100 for 24 h and were cryoprotected in 30 % sucrose before freezing. 15 μm serial sections were blocked with 5 % BSA containing 0.1 % TritonX-100 in PBS for 1 h at room temperature, and then incubated for 18 h at 4 °C with a rabbit anti-tyrosine hydroxylase polyclonal antibody (Merck-Millipore, Dundee, UK, catalogue number AB152) diluted 1:200 in PBS with 1 % BSA together with or without anti-GDF5 (R&D systems, 1:50 or Biopharm GmBh, 1:100). The sections were washed and incubated with appropriate secondary antibodies (Alexa-Fluor, Invitrogen, 1:500). The density of tyrosine hydroxylase-positive sympathetic fibers was determined as described [26]. All imaging and quantification was carried out blind.

### Whole-Mount preparations

Paraformaldehyde fixed tissue was processed to label tyrosine hydroxylase-positive sympathetic fibers by DAB-HRP staining followed by clearing in benzyl alcohol:benzyl benzoate as described previously [26]. To compare the extent of sympathetic nerve branching, a modified line-intercept method was used. Using ImageJ, a grid of 24 squares (4 × 6 squares, 158 μm side length per square) was aligned in a standard orientation on images of the trachea using the lateral axonal bundle entering the trachea as a guide. The number of fibre bundles intersecting the sides of squares in the grid was scored blind. Fibre density was estimated using the formula πDI/2, where D is the interline interval and I the mean number of intersections along one side of each square in the grid.

### Quantification of neuron number in the SCG

Estimates of the numbers of neurons in the SCG of wild type and *Gdf5*^bp^ mice were carried out by stereological analysis of 8 μm serial sections of the ganglia as described [25].

### Western blotting

This was carried out as described [43]. Extracted proteins were transferred to PVDF membranes which were blocked with 5 % dried milk in PBS with 0.1 % Tween-20 and were incubated overnight with anti-phospho-Smad1/5/8 (1:1000; Cell Signaling), anti-β-III tubulin (1:10000; Promega) or anti-ERK1/2 (1:1000; Cell Signaling). The appropriate peroxidase-linked secondary antibody (1:2000; Promega) was used to detect each primary antibody on the blots and staining was visualized using ECL plus (Amersham).

## Abbreviations

BMPR: bone morphogenetic protein receptor
BSA: bovine serum albumen
DRG: dorsal root ganglion
GDF5: growth differentiation factor-5
NGF: nerve growth factor
NT3: neurotrophin-3
PBS: phosphate buffered saline
SCG: superior cervical ganglion
TGFβ: transforming growth factor beta
